# Dangerous Liaisons: Long-Term Replication with an Extrachromosomal HPV Genome

**DOI:** 10.3390/v13091846

**Published:** 2021-09-16

**Authors:** Alix Warburton, Ashley N. Della Fera, Alison A. McBride

**Affiliations:** Laboratory of Viral Diseases, National Institute of Allergy and Infectious Diseases, National Institutes of Health, Bethesda, MD 20892, USA; alix.warburton@nih.gov (A.W.); ashley.dellafera@nih.gov (A.N.D.F.)

**Keywords:** HPV, papillomavirus, integration, DNA damage response, replication, fragile sites, partitioning, enhancers

## Abstract

Papillomaviruses cause persistent, and usually self-limiting, infections in the mucosal and cutaneous surfaces of the host epithelium. However, in some cases, infection with an oncogenic HPV can lead to cancer. The viral genome is a small, double-stranded circular DNA molecule that is assembled into nucleosomes at all stages of infection. The viral minichromosome replicates at a low copy number in the nucleus of persistently infected cells using the cellular replication machinery. When the infected cells differentiate, the virus hijacks the host DNA damage and repair pathways to replicate viral DNA to a high copy number to generate progeny virions. This strategy is highly effective and requires a close association between viral and host chromatin, as well as cellular processes associated with DNA replication, repair, and transcription. However, this association can lead to accidental integration of the viral genome into host DNA, and under certain circumstances integration can promote oncogenesis. Here we describe the fate of viral DNA at each stage of the viral life cycle and how this might facilitate accidental integration and subsequent carcinogenesis.

## 1. Human Papillomaviruses and Their Association with Human Disease

**Figure 1:**
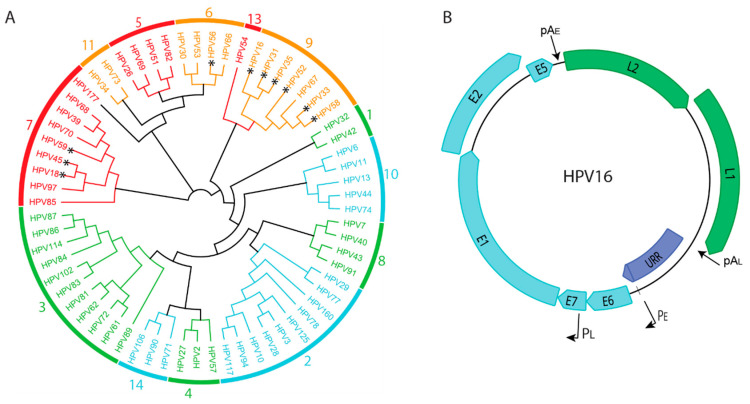
(**A**). Phylogenetic tree derived from the E7 protein sequences of human Alphapapillomaviruses. High-risk viruses are shown in red/orange and low-risk viruses in green/blue. Viral species are shown on the perimeter of the tree. Those viruses that have been found to be frequently integrated are indicated with an asterisk * (Viral Integration site database. https://bioinfo.uth.edu/VISDB ; accessed on 10 August 2021). (**B**). Map of the HPV16 genome. Early genes are shown in cyan and late genes in green. The Upstream Regulatory Region, URR, is shown in purple. Also indicated are the early and late viral promoters, PE and PL, and the early and late polyadenylation signals, pAE and pAL.

To date, 440 different human papillomaviruses (HPVs) have been described [1]. They are classified into five different genera that are named Alpha, Beta, Gamma, Mu, and Nupapillomaviruses. In general, HPVs from the Alpha, Mu, and Nu genera cause a wide range of cutaneous warts while HPVs from the Beta and Gamma genera are associated with asymptomatic infection of the skin [2]. Some Alphapapillomaviruses also infect the mucosal epithelium at the mucocutaneous junctions located at bodily orifices such as the mouth and anogenital tract. A subset of these HPVs is considered oncogenic, and persistent infection with these viruses can result in anogenital or oropharyngeal cancers [3]. Figure 1A shows a phylogenetic tree of Alphapapillomaviruses and indicates those which are considered low, or high, oncogenic risk.

## 2. Papillomavirus Genome Organization

All human papillomavirus (HPV) genomes are small, double stranded circular DNAs of about 7–8 kb (Figure 1B). The genome is assembled with host histones into a minichromosome that replicates in the nucleus of infected cells. The genome consists of three distinct regions: the upstream regulatory region, or URR; the early coding region; and the late coding region. The upstream regulatory region contains cis-regulatory elements such as promoters, enhancers, and the origin of replication. The early coding region contains genes expressed in the earlier stages of infection and the late coding region encodes the capsid proteins, L1 and L2. All papillomaviruses encode four core proteins: the early E1 and E2 proteins that are required for viral DNA replication and the late L1 and L2 capsid proteins. Many HPVs also contain genes for smaller, less well conserved proteins such as E5, E6, and E7. These accessory proteins are essential for immune evasion and for manipulating the cellular environment to support the viral lifecycle in a stratified host epithelium [4].

## 3. The Papillomavirus Infectious Cycle

The infectious cycle of papillomaviruses is quite resourceful. The virus accesses the self-renewing basal cells of a stratified epithelium through a micro-fissure or abrasion. The virus initially attaches to heparin sulphate proteoglycans on the exposed basement membrane at the site of wounding and then attaches to a receptor on the keratinocytes. The virus is internalized and traffics through the endocytic pathway to the peri-nuclear trans-Golgi network where it remains until the nuclear membrane breaks down during mitosis. At this point, the virion is mostly intact and enclosed in vesicles derived from endocytic membranes. The vesicles attach to mitotic chromosomes and are therefore retained in the nucleus after cell division. The virions are next observed associated with the PML nuclear bodies (PML-NBs) as the nucleus reassembles; this is likely the location of early viral transcription and replication.

The E1 and E2 proteins are expressed early in infection and replicate the viral DNA to a low copy number [5]. The genomes must become “established” as extrachromosomal minichromosomes that replicate in synchrony with the host chromosomes. This “establishment” process is facilitated by the E5, E6, and E7 accessory proteins that function to repress innate immune detection, suppress responses to the replication of foreign DNA, and promote proliferation of infected cells.

In a stratified epithelium, the basal cells can divide symmetrically to produce more basal cells, or asymmetrically to generate one daughter cell that remains in the basal layer and another that is pushed off the basement membrane and moves up through the stratified layers of the epithelium, acquiring characteristics of differentiation as it proceeds. This process ensures that a reservoir of infected cells is maintained in the basal layer while, at the same time, infected cells progress through the differentiation process and generate virus particles.

The strategy of restricting high levels of viral DNA production and protein synthesis to terminally differentiated cells helps the virus to evade immune detection. However, it also necessitates the synthesis of viral DNA in cells that would have normally exited the cell cycle and do not contain the factors required for DNA replication.

## 4. The Fate of Viral DNA through the Infectious Cycle

**Figure 2:**
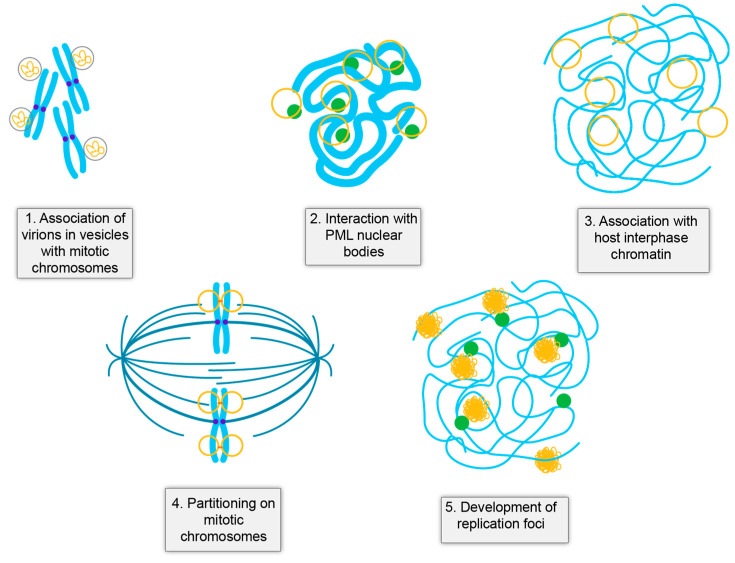
Close association of viral and host DNA in the HPV Infectious Cycle. Shown are five stages of the infectious cycle where there is a close juxtaposition of viral DNA (yellow) and host DNA (cyan). PML-nuclear bodies are shown in green, cohesin (orange), and the spindle (teal).

In persistent viral infection, the location of the viral genome at each stage of infection is critically important. At any step, the viral DNA could be lost from the cell, silenced by the host, or detected by innate immune pathways. HPV genomes exist as small extrachromosomal minichromosomes and their location at each stage of the infectious cycle seems to be precisely preordained to maintain low level infection while producing large numbers of progeny genomes in terminally differentiated cells. At many of these stages there is a close interaction between the viral and host genomes that could promote accidental integration at these sites (Figure 2).

### 4.1. HPV Virions Associate with Mitotic Chromosomes on Entry

Upon entry, the HPV virion is trafficked through the endocytic pathways and only enters the nucleus when the nuclear membrane breaks down in mitosis [6,7,8]. At this stage, the virion is still encased in a vesicle derived from endocytic membranes that helps evade immune detection [9,10,11,12,13]. A small peptide from the L2 protein projects through the vesicles and interacts with the still condensed mitotic chromosomes [11,13]. Little is known about the nature, or precise location, of this interaction with chromosomes.

### 4.2. Establishment of HPV Infection Occurs at PML Nuclear Bodies

Efficient establishment of papillomavirus infection requires the PML component of the PML-NBs [14,15,16]. This seems counterproductive because PML-NBs are best known for their role in antiviral defense, however many DNA viruses initiate replication at these sites, displacing or degrading the repressive components [17,18,19]. The PML bodies are dissociated in mitosis and must reform as the nucleus reassembles [20]. Correspondingly, Guoin and colleagues showed that PML is recruited to the viral genome even before it escapes the transport vesicle [21]. When expressed alone, the L2 protein localizes to the PML bodies and so it is likely that L2 mediates this initial association [19,21,22]. Other components of the PML bodies, such as Sp100, are also recruited to the reforming nuclear bodies but this is delayed until the genome is released from the virion [21]. Several isoforms of the Sp100 protein repress early transcription and replication [16], however, in turn, L2 can displace Sp100 from PML bodies in an attempt to counteract host defenses [19].

PML nuclear bodies are linked to specific regions of host chromatin and are associated with DNA repair processes and interferon signaling [23,24,25]. This is likely to be the first time in the infectious cycle that the viral and cellular genomes are in close proximity. Viral DNA must undergo a few rounds of unlicensed DNA amplification to establish a low copy number of viral genomes in the cell nucleus, and the PML bodies might be a permissible nuclear location to initiate infection. A common theme in nuclear architecture is that nuclear bodies sequester proteins and concentrate biochemical reactions, and this could be beneficial for a viral genome attempting to initiate infection [25].

### 4.3. Establishment of Infection

The phase of infection between the initial viral transcription and replication at the PML bodies and the generation of an infected cell that maintains extrachromosomal viral genomes is termed “establishment”. This phase can be quantitated using a colony forming assay that measures the ability of transfected (or infected) oncogenic HPV genomes to generate an immortalized cell colony in primary keratinocytes [16,26,27]. Establishment is a rare and inefficient event as the viral DNA needs to set up a beneficial location in the nucleus, escape epigenetic silencing, and evade both intrinsic and innate immune recognition of foreign DNA. For example, the efficiency of establishment is increased when restriction factors, such as Sp100, are downregulated [16].

### 4.4. The Maintenance Phase of Infection

The maintenance phase occurs in the proliferating basal cells of an infected epithelium. This phase can be studied in cell lines isolated from CIN1 (cervical intraepithelial neoplasia grade 1) lesions [28,29] that maintain the viral genome as a low copy number, extrachromosomal plasmid, or in immortalized cell lines obtained by transfection of oncogenic HPV genomes [30]. The viral genome copy number per cell can range from very low and undetectable in basal cells [31] to ~50–100 copies in cell lines [29]. The viral genomes are associated with host chromosomes in mitosis, and most likely also associate with host chromatin throughout the cell cycle. This association was first demonstrated with a bovine papillomavirus (BPV1) genome [32,33] but has been more difficult to observe with the lower copy number HPV genomes. Similar to Epstein-Barr virus and Kaposi’s sarcoma-associated herpesvirus (Gammaherpesviruses), the HPV E2 protein binds to sites in the viral genome and tethers them to host chromatin [34]. BPV1 and HPV1 E2 proteins bind in complex with the cellular Brd4 (Bromodomain-containing protein 4) protein to both transcriptionally active regions of host chromatin as well as to regions of chromatin undergoing replication stress (common fragile sites) [35,36,37,38]. Brd4 binds acetylated chromatin and is associated with many processes in the HPV infectious cycle [39].

Association with host mitotic chromosomes can fulfill several important functions for an extrachromosomal viral genome. Hitchhiking on the host chromosomes can partition the genomes, in equal numbers, to daughter cells to ensure that they are retained in the nucleus and do not trigger innate immune responses to cytoplasmic DNA [34,40]. The target region of attachment is also important as this could help promote viral transcription and replication activities but could also influence accidental integration of the viral genome.

**Figure 3:**
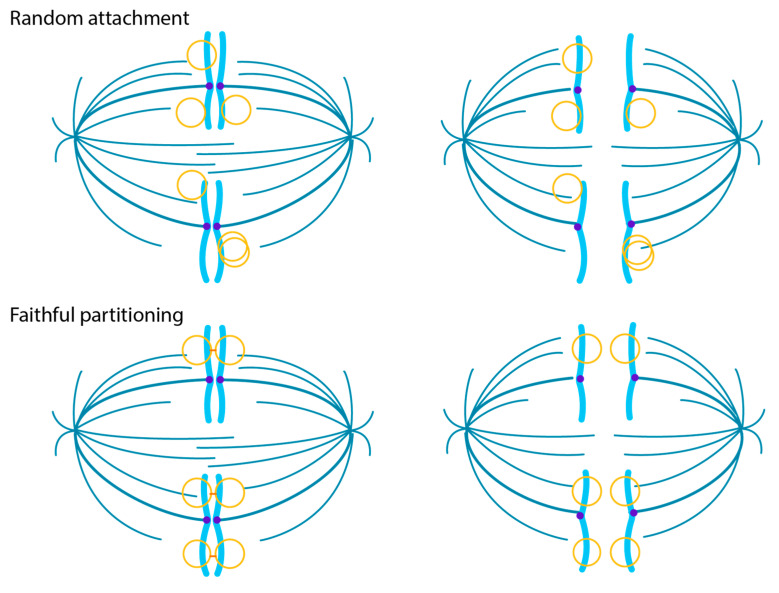
Models of viral genome partitioning. In the Random Attachment model, HPV genomes associate with the host chromosomes after DNA replication. In the Faithful Partitioning Model, the viral genomes are attached to the host chromatin throughout the cell cycle. Each daughter molecule is faithfully distributed to a daughter cell. Shown are viral DNA (yellow), host DNA (cyan), cohesin (orange), and the spindle (teal).

There are several ways in which extrachromosomal genomes can be partitioned to daughter cells in mitosis [34]. With sufficient genome copy number, untethered viral genomes could be randomly distributed to daughter cells, but this strategy could leave genomes in the cytoplasm when the nuclear membrane reforms. Alternatively, genomes could be transiently attached to a dynamic element such as the mitotic spindle or centrosome. Attachment to host chromosomes/chromatin is an efficient strategy that can provide additional benefits for the virus as it can capitalize on chromatin processes associated with the target region. As shown in Figure 3, The genomes can be partitioned in a semi-faithful or faithful manner: in semi-faithful partitioning, the viral genomes associate with host chromosomes after DNA replication; in faithful partitioning, the viral genomes are associated with specific regions of chromatin and replicate in situ, with each daughter genome segregating to a daughter cell in concert with the host chromosome. These partitioning strategies could pre-dispose the virus to accidentally integrate at the targeted regions. Sites of recurrent integration, known as integration hotspots, are associated with fragile sites and transcriptionally active chromatin [33,34] and could denote viral tethering sites within the host genome.

### 4.5. Viral DNA Amplification in Differentiated Cells

Differentiation of infected cells triggers high level expression of the E1 and E2 replication proteins, initiating amplification of viral genomes. The differentiated cells are in a G2-like phase of the cell cycle [41,42] and cannot replicate viral DNA by S-phase replicative mechanisms. Instead, the viral E7 and E1 proteins induce a DNA damage response that can amplify viral DNA in nuclear foci [43,44,45,46,47]. Many factors associated with homologous recombination are recruited to these nuclear foci, indicating that viral replication is mediated by recombination-directed replication processes [43,48,49,50,51,52,53]. Jang et al. observed that HPV replication foci were frequently located close to the same regions of the host genome that were targeted by the E2 and Brd4 proteins on mitotic chromatin [36]. This implies that the viral genomes remain associated with specific regions of host chromatin throughout the infectious cycle and initiate the formation of replication foci at these sites. Of note, these regions often overlap common fragile sites, which are regions of the genome that are difficult to replicate and undergo replication stress [36,54]. These regions could be beneficial to a virus that hijacks cellular DNA repair processes. However, they could also be at high risk for unintentional viral integration, as both viral and cellular replication and repair processes are closely juxtaposed. It is unlikely that integration of the viral genome in the productive phase of the infectious cycle would have much consequence for the host, but if similar processes or interactions occurred in proliferating cells, these processes could result in cells with integrated viral genomes.

## 5. Modes of Viral DNA Replication

The basic requirements for papillomavirus replication have been well defined and characterized. Replication is initiated at the replication origin in the viral genome, which contains specific binding sites for the viral E1 and E2 proteins [55,56]. E1 is a helicase that binds and unwinds the origin, allowing the cellular replicative machinery to synthesize viral DNA. E2 functions as a helicase loader by co-operatively binding to the replication origin with E1. The viral DNA undergoes DNA amplification at the initial stage of infection and at the late stage in differentiated cells, and this requires both E1 and E2 proteins. In addition, E2 supports maintenance replication by tethering the viral genome to host chromosomes [32,33,57].

During the maintenance phase of replication, the expression and nuclear location of the E1 protein is tightly regulated. Nuclear expression of E1 can induce a DNA damage response and cell cycle arrest [45,46,47]. The N-terminal domain of E1 contains multiple phosphorylation sites that regulate nucleocytoplasmic shuttling and ensure that E1 is retained in the cytoplasm until needed [58,59]. There are also reports that E1 is not always required for maintenance replication [60,61,62]. Likewise, it appears that the replication of HPV genomes is licensed (each molecule replicates once per cell cycle) in some cases, but not in others, and this correlates with E1 expression [63]. It is conceivable that E1 is only expressed sporadically in cells that maintain the viral genome and this could result in transient and limited DNA amplification that could potentially promote accidental integration of the viral DNA into the host genome.

Viral DNA is replicated using a bidirectional theta mode at early stages of replication, but in differentiated cells this changes to a unidirectional mode, most likely recombination-directed replication [52,64]. High levels of E1 and E2 proteins are expressed in differentiated cells to amplify the viral DNA [65,66,67], and HPVs activate both ATM (ataxia-telangiectasia mutated) and ATR (ATM and Rad3-related) DNA damage response pathways to support the synthesis of viral DNA at nuclear replication foci in differentiated cells [44,68]. Many factors associated with the DNA damage response are recruited to the viral replication foci [43,49,53,68,69,70]. Constitutive activation of the ATM and ATR pathways also results in DNA breaks in both viral and cellular genomes [53]. These breaks are repaired by the cellular repair machinery, but the juxtaposition of the viral and host DNA could promote accidental recombination of host and viral DNA. In fact, the HPV replication foci that form in differentiated cells are often located next to regions of host chromosomes undergoing replication stress [36]. These regions are related to common fragile sites, and it has been noted that HPVs frequently integrate in these unstable regions [36,71,72,73,74,75].

## 6. Unique Functions of Oncogenic HPVs

The E5, E6, and E7 proteins support the differentiation-dependent life cycle of HPVs by manipulating the balance of cellular proliferation and differentiation, and by antagonizing innate immune pathways [4]. Although all HPVs have similar strategies, the precise way in which certain HPVs exploit host pathways is different and can promote genetic instability and oncogenesis [76,77,78,79].

### 6.1. HPV-Mediated Regulation of Cellular Proliferation and Differentiation

The E6 and E7 proteins support the viral infectious cycle by promoting cell cycle entry, delaying differentiation, and preventing oncogene-induced senescence and apoptosis. The oncogenic HPVs do this by degrading the cellular p53 and retinoblastoma protein (pRb) tumor suppressor proteins [3]. The E6 proteins also degrade certain PDZ proteins (postsynaptic density protein, disc large tumor suppressor, zonula occludens-1 domain-containing proteins) [80]. These proteins regulate asymmetric cell division and epithelial cell polarity and, consequently, their degradation disrupts this equilibrium. The E6 protein also prevents replicative senescence by inducing expression of human telomerase reverse transcriptase (hTERT) to stabilize the erosion of telomeres [81]. Persistent HPV-mediated manipulation of these factors renders cells susceptible to mutation and genomic instability.

### 6.2. Evasion of Foreign DNA Recognition in HPV Infected Cells

The long-term differentiation-dependent strategy of HPV infection is very successful but requires evasion from host immune defenses to sustain a persistent infection. Human cells have multiple mechanisms to detect and eliminate foreign DNA, but HPVs have multiple tactics to avoid this.

Upon cell entry, HPVs traffic to the nucleus encased in membrane vesicles [9,10,11,82], Figure 2. The vesicle encased virions enter the nucleus when the nuclear membrane breaks down in mitosis and become attached to the condensed host chromosomes through a small peptide of the L2 protein that protrudes through the membrane vesicle [6,7,13,83]. This unusual strategy ensures that the viral DNA is retained in the nucleus when the nuclear membrane reforms and escapes recognition by cGAS/STING [12]. Another unusual feature of HPV genomes is that they are assembled into nucleosomes at all stages of infection [84]. This association provides an additional layer of regulation of gene expression and helps condense the viral genome for packaging. However, nucleosomes also inhibit recognition of DNA by cGAS/STING [85,86,87].

Like many DNA viruses, HPVs initiate infection at the PML nuclear bodies despite the fact that these bodies contain many interferon-responsive anti-viral factors [17]. Factors such as Sp100 recognize viral DNA and restrict HPV infection [16,21,88]. However, in turn, HPVs reorganize the bodies and displace repressive factors [19].

Little is known about evasion of foreign DNA detection during the maintenance phase of replication, although the nuclear DNA sensor IFI16 can repress viral transcription and replication by epigenetic silencing [89]. In dividing cells, tethering of viral minichromosomes to host chromosomes can also ensure that viral DNA is retained in the nucleus, thus preventing activation of cytoplasmic DNA sensors such as cGAS/STING [40].

The differentiation-dependent life cycle strategy also prevents immune recognition as high levels of viral DNA replication and transcription occur only in cells that are destined to die and are not under strict immunosurveillance. Nevertheless, factors such as Sp100 associate with viral DNA in late replication foci and restrict late viral transcription and replication. This engagement of Sp100 with viral DNA can also be observed in HPV16-infected cervical lesions [90].

In addition to the evasion tactics described above, the E5, E6, and E7 proteins inhibit detection of viral DNA by interfering with foreign DNA sensors, cytokine production, and interferon signaling pathways [91,92,93]. Therefore, the genomes of persistently infected cells replicate alongside viral DNA that would be normally eliminated from the cells. This environment of reduced immune surveillance and disrupted tumor suppressor pathways (that eliminate cells with damaged DNA) most likely promotes the inadvertent integration of viral DNA into the host genome.

## 7. HPV Genome Integration

### 7.1. Integration Is Frequent in HPV Associated Tumors

HPV DNA is found integrated in many HPV-associated anogenital and oropharyngeal cancers, but this is not part of the viral life cycle. Instead, this occurs by accident and, in some cases, can drive oncogenesis. In fact, >80% HPV-positive cervical carcinomas in The Cancer Genome Atlas have integrated HPV DNA [94]. However, not all cancers contain integrated HPV DNA, and some contain extrachromosomal genomes, or a mix of integrated and extrachromosomal DNA [95,96,97,98,99].

Likewise, not all integrated HPV genomes drive oncogenesis. There are often multiple HPV integration sites in a single tumor but most often only one is transcriptionally active while others can be considered silent passenger integrants [94,100,101]. Integration probably occurs frequently, particularly in a genetically unstable environment, but only those integration events that provide cells with a selective growth advantage drive clonal expansion and oncogenesis [102,103]. In fact, MmuPV1 (Mus musculus papillomavirus 1) genomes are frequently integrated in benign mouse tumors [104].

### 7.2. Structure of the HPV Genome at Integration Sites

**Figure 4:**
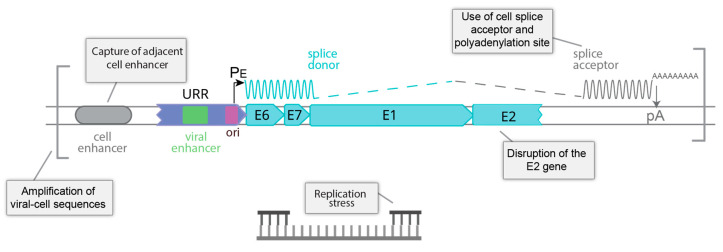
Factors influencing expression of an integrated oncogenic HPV genome. Shown are five factors that can influence whether HPV genome integration sites dysregulate expression of the E6 and E7 oncogenes and drive carcinogenesis.

Cancers associated with the oncogenic human Alphapapillomaviruses require expression of the E6 and E7 oncogenes for continued growth; abrogation of E6/E7 gene expression reactivates tumor suppressor pathways and induces growth arrest and senescence [105,106]. Concordantly, only the E6 and E7 genes are consistently present and transcribed from integrants that drive oncogenesis; however, this expression is dysregulated, resulting in aberrant proliferation, disrupted cell cycle checkpoints, and progressive genetic instability. In most cancers with integrated HPV genomes, E6 and E7 are expressed from a transcript that initiates at the early promoter (PE) in the upstream regulatory region but is spliced from a viral splice donor to a splice acceptor in the adjacent cellular DNA [107]. A cellular polyadenylation site is also required and the resulting hybrid transcripts are often more stable than their viral counterparts [108]. A typical viral integrant is shown in Figure 4.

Another way in which E6/E7 oncogene expression can be dysregulated is by disruption of the E2 gene. The E2 protein represses the viral early promoter and so integration in E2 can alleviate this repression and dysregulate viral oncogene expression [109]. Methylation of the E2 binding sites in the promoter/origin region can also alleviate E2-mediated repression, even in extrachromosomal genomes [110,111].

HPV integration sites can be classified as Type 1, 2, or 3, depending on the number of genomes integrated at a single locus [103,112,113]. In Type 1, a single genome is present and in type 2, multiple tandem repeats of head-to-tail viral DNA are integrated at a single genomic locus. Type 3 sites also contain multiple tandem copies of the viral genome, but they are interspersed with repeats of flanking cellular DNA [112,114]. Several models have been proposed to explain how these repeats are generated: for example, endoreduplication of the integration site and flanking sequences, followed by unequal recombination could result in tandem repeats of viral-host sequences [115]. Viral-host DNA concatemers could also be generated by a transient loop that functions as a template for rolling circle replication [116]. There is abundant evidence for the existence of long stretches of tandem viral-host repeats in the genome sequences of HPV-associated cancers, however, it has also been proposed that these tandem repeats can occasionally exist as large extrachromosomal circular DNA (ecDNA) molecules [96,99,117].

At early stages of integration, E1 and E2 (expressed from extrachromosomal genomes in the same cell) could potentially initiate multiple rounds of “onion skin” replication and induce focal genomic instability at the integration site [118,119]. Alternatively, as described below, integration into regions of the host genome undergoing replication stress could also promote amplification around the integration site [120]. As described above, aberrant expression of E1 can induce DNA damage and growth arrest [45,46]. Thus, integration that eliminates E1 expression could provide a further, selective growth advantage.

### 7.3. Landscape of the Cellular Genome at HPV Integration Sites

Overall, it is thought that the viral genome integrates at random sites in the human genome. However, analysis of many integration sites has identified some preferential regions that are likely related to the nuclear location of the viral DNA at different stages of the viral life cycle.

HPV genomes are often integrated in transcriptionally active regions of open chromatin and these events probably reflect the association of the extrachromosomal viral minichromosome with such regions [35,38,121,122,123]. Somatic copy number amplifications are common at HPV integration sites and can influence local gene expression [124]. The cellular enhancers in transcriptionally active regions of host DNA at integration sites can promote viral oncogene expression and, in some cases, co-amplification of the viral-host sequences can result in enhancer capture and generation of a viral-host super-enhancer [112,114]. The chromatin adapter protein, Brd4, is highly enriched in cellular enhancers and super-enhancers and plays important roles throughout the viral life cycle [39]. Super-enhancers regulate transcriptional hubs important for cell identity [125,126] and could provide an attractive nuclear location for HPV minichromosomes. Our analysis of HPV integration sites in cervical cancer reveals integration hotspots at Brd4-enriched transcriptional regulatory hubs [101]. To date, most studies have analyzed linear viral-host sequences at integration hotspots, but rapid advances in three-dimensional genome interactions should provide further insight into how HPV integration perturbs host gene expression [127,128].

Another class of HPV integration hotspots are common fragile sites [73,74]. Common fragile sites are regions of the host genome that have difficulty completing replication and frequently undergo replication stress, and this is often due to clashes in replication and transcription that occur at large genes [54]. We have previously shown that a complex of the viral E2 protein and host Brd4 protein binds to regions of the host genome that overlap common fragile sites [36]. Furthermore, replication foci frequently form at these same regions in differentiated cells [36]. HPV genomes may tether to these regions because of the enrichment of Brd4, but the susceptibility of these regions to undergo replication stress and the dependence of HPV replication on the DNA damage response could enhance accidental integration at these sites. Resulting clonal selection of integrated HPV genomes at sites of recurrent integration likely reflects the high level of transcription at such regions.

Figure 4 outlines the processes that could promote oncogenesis at an HPV integration site.

## 8. Conclusions

Papillomaviruses have evolved a remarkable life cycle where they manipulate key cellular pathways at every stage of infection. In most cases, infections are self-limiting and are minimally detrimental to the host. However, one small subset of HPVs infects the mucosal epithelia and has evolved to disturb the balance of proliferation and differentiation, and to disrupt innate immune defenses, in such a way as to promote genomic instability of the host. The close association of viral and host chromatin processes further increases the probability that the viral DNA accidently integrates into the host DNA, forming a stable association that can drive oncogenesis.

## Data Availability

Not applicable.
